# Balance and Muscle Strength in Elderly Women Who Dance Samba

**DOI:** 10.1371/journal.pone.0166105

**Published:** 2016-12-01

**Authors:** Marcos Maurício Serra, Angelica Castilho Alonso, Mark Peterson, Luis Mochizuki, Júlia Maria D'Andréa Greve, Luiz Eugênio Garcez-Leme

**Affiliations:** 1 Laboratory for the Study of Movement, Institute of Orthopedics and Traumatology, School of Medicine, University of São Paulo, São Paulo, Brazil; 2 Department of Physical Medicine and Rehabilitation, University of Michigan Health System, Ann Arbor, Michigan, United States; 3 Departament of Pos Graduate Program in Aging Science, São Judas Tadeu University (USJT), São Paulo, Brazil; University of Lethbridge, CANADA

## Abstract

Considering the growth of the aging population, and the increasing risk for falls and related morbidity, it is vital to seek efficient, comprehensive, and culturally relevant prevention programs for elderly people to reduce risks for falls. The aim of the present study was to evaluate the postural balance and muscle strength among women participating in the "Wing of Baianas" in the carnival parades. One hundred and ten women, with an average age of 67.4±5.9 years, were divided into two groups: Baianas group—elderly participants of the carnival parades in the “Wing of Baianas”, and a Control group of women who do not dance samba. Assessments included a physical activity questionnaire, isokinetic muscle strength testing for the knee extensors and flexors, and a postural balance assessment completed on a force platform. There were no differences between groups, for postural balance outcomes, during the eyes open condition; however, with eyes closed, there was a significant effect between groups (Baianas vs Control) in all variables. The Baianas group showed less medio-lateral displacement (*p* < 0.04); and anteroposterior displacement (*p* < 0.007); larger amplitudes of medio-lateral displacement (*p* < 0.001); and anteroposterior displacement (*p* < 0.001); increased mean velocity (*p* < 0.01); and elliptical area (*p* < 0.01) There were no differences in the isokinetic peak torque corrected by body weight, total work and flexor/extensor ratio. Participation in the Wing of Baianas is associated with better balance with closed eyes, but there were no differences between dancers and non-dancers for muscle strength.

## Introduction

Similarly to other developing countries, brazilian elderly population is growing (20.6 million older than 60 years old, and three million older than 80 years old). In Brazil, more life expectancy (67.3 years old for men and 75.2 years old for women) [1] will take the elderly up to be 32 millions by 2025. As this population grows the pressure for proper health services increases. Health-related problems emerge as important issues to be addressed by researchers in order to provide solutions to improve and promote better health services.

The elderly fears an accidental fall because it can break bones or hurt, kill or lead to physical or psychological constraints [2]. Falling also is associated with frailty or acute illness; thus, it is a serious public health burden [1, 2]. Neuromuscular deficits (e.g., sarcopenia) due to aging impair physical performance, reducing muscle strength and power performance and balance [3, 4]. It is important to evaluate postural control and associate it to the risk to fall. Maki et al. [3] showed that medio-lateral postural sway amplitude (blindfolded conditions) is the single best fall predictor.

Static and dynamic activities to improve postural balance are provided in controlled environments. Balance rehabilitation programs are based on activities such as walking in a straight line, one foot support, walking on different types of surfaces, and overcoming obstacles [1,3,5]. Sport and other cultural activities are used in rehabilitation programs [6]. Tradicional dance activities improve balance [6, 7]. However, it is not clear whether folkloric dance improves muscle strength and postural balance [6]. Turkish folk dance [7], greek dance al. [8], thai dance [9] may change and improve performance in balance tests. Those studies did not evaluated lower limb muscle strength. Samba dance is a brazilian typical musical rhythm and it is largely presented during the brazilian carnival parades. Those parades are organized in blocks, usually called wings. Associations named samba schools are responsible to prepare and show such parades, under a specific song theme and its related choreography and costumes. Whatever theme is choosen one mandatory wing is "Wing of Baianas". Only older women, named the Baianas, participate into this traditional wing and their costumes are usually the same (long and large-structured dresses).Their traditional choreography involves walking to several directions, spinning around the coronal axis and transferring the body weight from one leg to the other. Although empirical or anecdotal statements may be expressed to support the use of samba dance as an activity to improve balance control, there is no evidence about it. Therefore, what is Baianas' performance in balance tests?

Cultural and social aspects of samba are deeply studied but its impact to health and quality of life is unknown. To promote adherence in physical activity programs, such activities should incorporate participant’s lifestyle, to have cultural context and respect cultural traditions, in order to improve its values and needs of the person [7]. Baianas have such feelings when they participate into that wing. Can we believe that samba dance, in special, what Baianas do, could be applied into a balance training to reduce the risk to fall?

We want to find answers to those two questions. Thus, the aim of the present study was to evaluate the postural sway and muscle strength in women who participates into the Wing of Baianas in the carnival parades. We will compare Baianas’ performance in balance and muscle strength tests with matched control group with women who do not dance samba. Our hypothesis is that Baianas will show less postural sway and more muscle strength than control group.

## Materials and Methods

### Study location and ethical issues

This is a controlled cross-sectional study conducted at the Motion Study Laboratory of the Institute of Orthopedics and Traumatology, Hospital das Clinics, University of São Paulo School of Medicine. All participants gave their written informed consent to participate in this study, which was approved by the Ethics Committee of the Medical School at University of São Paulo (number 723/2009).

### Participants

Population is older women who participate into Wing of Baianas. Pilot study with 17 older women was used to calculate the sample size. For 0.80 statistical power and 5% level of significance, it would be necessary to have at least 55 participants in each group. Then, participants were one hundred and ten elderly women, divide into Baianas group (older women who were dancers from two traditional Schools of Samba in São Paulo, Brazil) and Control group (older women living at the nearby community of samba schools who did not attend to samba or other types of dance). Participants in both groups were age matched. Inclusion criteria for both groups were: 1) absence of vestibular, proprioceptive, auditory, or neurological impairment, and/or of any mental disturbances or disorders; 2) no use of medications that might compromise postural balance; 3) absence of lesions, surgery, or disease that might have caused lower-limb joint limitations over the previous six months; 4) absence of any lower-limb dysmetria; 5) presence of clinically normal gait, without claudication, 6) performance in Mini Mental State Examination (MMSE) within normal values for education. For Baianas group only, there were also these inclusion criteria: 1) participation in the Wing of Baianas for at least two carnivals; 2) regular attendance/participation in the choreography rehearsals of Baianas once or twice a week, for five months during the period before Carnival; and 3) no participation in other physical activities. For the Control group only, others inclusion criteria were no involvement in the carnival parades, with no regular participation in physical activities.

### Baianas dance

Baianas sing the samba school theme song while they are dancing. This samba song is related to samba school they are attending. The choreography of this carnival dancing is based on drums rhythm, turns and spins, and body weight transfers between feet. They usually turn and spin to right or left as they move forward. They balance their gait leaving the body weight upon one or two feet. During the chorus, they perform several spins. Baianas start to train their dancing at least five months before carnival. They do it twice a week for one hour. During training phase, they wear regular clothes; while during carnival paride, they wear 10–25 kg weigth costumes.

### Measures

Participants informed their age, current health and medical history, limb dominance, it was measured body mass and height, and it was calculated body mass index (BMI). Participants were also asked about how many falls they had in the last 12 months. Participants had 10.8 years experience in dancing samba. IPAQ (International Physical Activity Questionnaire short version) [10] was applied to characterize the status of participants’ physical activity level, which is classified as: a) **Very active** for individuals who practice activities five days a week or more with 30 minutes per session; b) **Active** for individuals who engage in physical activities ≥ 150 minutes per week; c) **Irregular physically active** for individuals who engage in physical activity, but not enough to be classified as **Active** as they do not meet the recommendations for frequency or duration of activity. **Irregular physically active** persons are divided into: **Irregular physically active A**: for individuals who achieve at least one of the recommended criteria for frequency or duration of activity; and **Irregular physically active B**: for individuals who do not meet any of the recommended criteria for frequency or duration of activity; d) **Sedentary** for individuals who do not carry out any physical activity for at least 10 continuous minutes per week [10]. All the tests lasted approximately one and a half hour.

### Postural balance assessment

Postural balance assessment (posturography) was performed on a mobile force platform (AccuSway Plus, AMTI, MA, USA). The force platform was connected to a signal-amplifying interface box (PJB-101) that was linked to a computer by means of an RS-232 cable. Data were recorded and stored with Balance Clinic software. Sampling frequency was 100 Hz and raw data was filtered with fourth-order Butterworth filter with 10 Hz cutoff frequency. Center of pressure (COP) for anterior-posterior (AP) and medio-lateral (ML) directions was calculated from ground reaction forces (F_i_) and moments of force applied (M_i_) onto the force plate according to Eqs 1 and 2:

$$COPap=\frac{M_{ML}+F_{AP}.d}{F_{V}}$$ (1)

$$COPml=\frac{M_{AP}+F_{ML}.d}{F_{V}}$$ (2)

Where d is the thickness of the plate.

Standard position during tests was quiet standing posture, parallel feet with width as wide as the hip width, arms with extend elbows along the trunk, and eyes looking straight ahead at a target 5 m away. The place where to lie the feet was drawn on a sheet of paper and glued onto the force platform. The reference points to draw this figure was distal hallux phalanx, fifth metatarsal head, and lateral and medial malleolus for each foot. Three measures were made for eyes opened condition (EO) and other three for eyes closed condition (EC). Each trial last 60 seconds.

### Isokinetic dynamometry

Knee extension and flexion strength was measured with isokinetics dynamometry (Biodex Multi-joint System 3, Biodex MedicalTM, Shirley, NY, USA). The isokinetics machine was calibrated 30 minutes prior the test. One-minute after standardized warm-up (four submaximal repetitions to become familiar with the equipment), participants performed concentric extension and flexion knee movements at 60°/s. Standard initial position was sitting with hips at 90° of flexion and participant was holder with sitting belts. Two sets of five maximal repetitions of knee extension and flexion starting with the dominant limb were performed. Continous verbal encouragement was given during trials to promote maximum effort during actions. Participants had one minute rest interval between sets to avoid fatigue.

### Variables

Balance variables were calculated from COP time series: it was calculated the root mean square (RMS), amplitude, mean velocity, and the elliptical area encompassing 95% of COP [11, 12]. Muscle strength variables were calculated from isokinetics tests. Only torque data from the second set was used for analysis to prevent a confounding influence of motor learning on clinical isokinetic performance [13]. The isokinetic variables used were maximum peak torque normalized by body weight (PTQ/BW), total work (TW), and torque knee flexor/torque knee extensor ratio.

### Statistical analysis

Continuous data are presented with means, standard deviations or medians. Data distribution was evaluated with Komogorov-Smirnov test. Anthropometric variables, physical activity level, number of falls and balance variables had normal distribution and analysis of variance were applied to compare groups. Mann Whitney U test was used to compare muscle strength variables between groups. Data were analyzed with SPSS 20.0 software. Level of statistical significance was set at 5%.

## Results

Average and standard deviation data related to demographic information is in Table 1. Only height was different between groups. Control group is taller (*p* = 0.03) than Baianas group. Age, body mass, BMI, number of falls and MMSE were similar between groups. Every women were classified as irregular physically active A or B, and none of them met the minimum recommendation for exercise participation.

**Table 1:** Average and standard deviation (SD) data about demographic information of participants.

	Baianas group	Control Group
**Age (years)**	66.8(6.2)	68.1(5.5)
**Body mass (kg)**	72.7(13.3)	69.3(12.1)
**Height (cm)**	157(0.05)	154(0.06)*
**BMI (kg/m**^**2**^**)**	29.3(5.0)	28.9(4.6)
**MMSE (point)**	26.4(3.7)	27(3.0)
**Physical activity (IPAQ) %**		
**Irregular activity A**	23.6	18.1
**Irregular activity B**	76.4	81.9
**Report of falls in the past year %**		
**Yes**	20	21.8
**No**	80	78.2

Student t test

* p = 0.03

Abbreviations: BMI, Body Mass Index; IPAQ, International Physical Activity Questionnaire; MMSE, Mini Mental State Examination.

Average and standard deviation of balance variable presented for opened eyes and closed eyes conditions are in Table 2. For eyes opened condition: group condition did not affect ML RMS (F_1.108_ = 0.04 *p* = 0.84); AP RMS (F_1.108_ = 3.0 *p =* 0.08), ML amplitude (F_1.108_ = 0.045 *p* = 0.83) AP amplitude (F_1.108_ = 3.6 *p* = 0.12); mean velocity (F_1.108_ = 0.1 *p* = 0.68) and COP area (F_1.108_ = 0.44 *p* = 0.50). For eyes closed condition, group condition affected all balance variables. Baianas group showed less ML RMS sway (F_1.108_ = 4.1 *p* = 0.04) and AP RMS (F_1.108_ = 3.3 *p =* 0.007); more ML amplitude (F_1.108_ = 13.5 *p* < 0.001) and AP amplitude (F_1.108_ = 11.6 *p* < 0.001); faster mean velocity (F_1.108_ = 8.4 *p =* 0.004) and larger COP area (F_1.108_ = 6.7 *p* = 0.01) (Table 2).

**Table 2:** Between group comparisons for postural balance in semi-static conditions with eyes open and eyes closed.

		Baianas Group Mean (sd)	Control Group Mean (sd)
**Eyes open**			
	**Medial-lateral RMS (cm)**	0.24(0.11)	0.23(0.13)
	**Medial-lateral amplitude (cm)**	1.27(0.52)	1.25(0.65)
	**Anteroposterior RMS (cm)**	0.40(0.15)	0.36(0.13)
	**Anteroposterior amplitude (cm)**	2.19(0.72)	1.93(0.71)
	**Mean velocity (cm/s)**	0.86(0.20)	0.88(0.29)
	**COP Area (cm**^**2**^**)**	1.92(1.48)	1.74(1.41)
**Eyes closed**			
	**Medial-lateral RMS (cm)**	0.25(0.12)	0.49(0.85)*
	**Medial-lateral amplitude (cm)**	1.40(0.56)	0.87(0.90)*
	**Anteroposterior RMS (cm)**	0.45(0.17)	0.68(0.90)*
	**Anteroposterior amplitude (cm)**	2.63(0.97)	1.73(1.69)*
	**Mean Velocity (cm/s)**	1.18(0.35)	0.89(0.65)*
	**COP Area (cm**^**2**^**)**	2.36(1.67)	1.60(1.37)*

*Differences between Baianas and Control groups at *p* < 0.05.

Abbreviation: RMS. root mean.

Muscle strength variables was similar between groups (Table 3).

**Table 3:** Between group comparisons for muscle strength of knee extensors and flexors between the Control group and the Baianas group.

		**Baianas Group Median**	**Control Group Median**	**P-(value)**^a^
**Dominant extensor**				
	PT/BW (%)	124.4	122.4	0.59
	Total Work (J)	306.4	290.3	0.94
	AGO/ANT	45.9	44.4	0.69
**Dominant flexor**				
	PT/BW (%)	54.5	56.5	0.83
	Total Work (J)	151.7	137.3	0.57
**Not Dominant extensor**				
	PT/BW (%)	127.5	124.9	0.73
	Total Work (J)	296.0	300.5	0.88
	AGO/ANT	46.1	44.8	0.33
**Non-Dominant Flexor**				
	PT/BW (%)	55.9	54.4	0.55
	Total Work (J)	141.5	138.1	0.31

^a^ P-value were calculated using the Mann Whitney U.

Abbreviations: PT/BW, Torque peak normalized by body weight; AGO/ANT, Agonist/antagonist ratio.

## Discussion

This study focused on hypothetical differences on postural control between older women who participate in samba parades and older women who do not. The aim of our study was to evaluate the postural sway and muscle strength in women who participates into the Wing of Baianas. Such women, called Baianas, presented less variable but larger and faster postural sway compared with controls during eyes closed condition. Under restriction of sensory information, importance of residual sensory information is enhanced and real capacity of the postural system to bump external and internal perturbations is tested. Our results suggest that postural control in Baianas have different behavior compared with older women without actual experience in samba dancing.

Baianas presented less variability in postural sway with eyes closed. Older adults showed more COP variability while leaning maximally forward compared with young adults. Less variability suggest less flexibility in postural sway [14]. However, more variability in extreme conditions, like Van Wegen et al. [14] suggest a bold or dangerous strategy. More bold actions (more variability and more amplitude in postural sway) increases the risk to fall. Young adults when they lean maximally presented less variability, similarly to Baianas group did when they have stood in quiet standing with eyes closed [14]. Our results suggest that Baianas might use a more secure strategy under sensory constraints.

Baianas presented larger postural sway with eyes opened. This result contradicts our hypothesis. We suggested that Baianas would show less balance sway compared with controls. This hypothesis is only supported under eyes closed condition. More postural sway is usually observed when sensory information is restricted [11, 12] when older adults are compared with young adults or when sedentary older adults are compared with physically active older adults [14]. Larger postural sway under an unstable condition might be mean a proper strategy to maintain balance [15]. It means that Baianas used the active search function for postural control and they can more prepared for unstable balance conditions compared with the controls.

Engage in dancing activities can be ease, fun and cheap, but lead to very specific changes. The high number of older adults in dancing activities led us to consider the role dance in health, particularly for postural balance and fall prevention [16]. Baianas and controls had similar cognitive performance in Mini mental test and similar muscle strength. Granacher et al. [17] and Alpert et al. [18] did not show any improvement in cognition after practicing dancing. Granacher et al. [17] did not find effect of salsa dancing in muscle strength. Muehlbauer et al. [19] showed that muscle strength and postural balance are not correlated, which supports the notion that balance and strength are separate components of neuromuscular function. The stimuli associated with “samba dance” may not have been sufficient to generate differences in strength between groups; however, it is also likely that the non-specific nature of the strength test was not sensitive enough to detect subtle differences in dynamic and functional strength performance between groups.

Balance variables were similar between Baianas and controls when eyes were opened. Zhang et al. [20] presented similar results when elderly performed movements in different directions and speeds, including spins. Hui et al. [21], with aerobic dancing in seniors, and Kattenstroth et al. [22] with semi-professional ballroom dancers, also presented similar results compared with controls. Many postural reflexes can be triggered by both the vestibular and the visual systems [11, 12]. Thus, vision can compensate for some loss of balance.

Baianas presented larger and faster postural sway when eyes were closed. This condition increases the risk to fall because postural sway becomes closer to the limits of basis of support and diminishes the time to change postural sway direction to avoid such boundaries. This is the definition of time to contact [15]. Kattenstroth et al. [23], in a functional reach test, showed that elderly presented more velocity and sway area. Baianas show a bold strategy, trained with several spins that might stimulate a kind of vestibular trainig, which may compensate the absence of visual information. Zhang et al. [20] showed that waltz turns in different directions continuously stimulates the vestibular system and improves balance.

The specific training to improve postural balance, performed in the traditional way (static and dynamic activities in controlled environments (in only one plane) such as walking in a straight line, decreased base of support, walking on different types of surfaces, and overcoming obstacles), has good results [6]. Variable motor experiences provided by dance, with free movements in different planes and axes, and with rotational movements of the head and the body seem to promote intense vestibular stimulation and therefore are less dependent on visual input for postural control [24]. The activities performed by Baianas can be considered a dual task. Baianas have to sing and dance, which could stimulate several mechanisms of postural control to improve performance in balance tasks.

Limitations and Prospects: Limitations of the study the absence of control in intensity of the training, which may have led to variations between the individuals in the Baianas group. Whether samba prevents falls, or minimizes potential deficiencies in postural balance, remains an open question for experimental studies.

The findings of this study demonstrated that individuals who participated in samba dance had superior postural sway with the eyes closed. Rehabilitation of postural balance could involve classical therapeutic exercises complemented with activities such as samba that involve motor improvisations (spins and turns). This combination seems to have a positive influence in the elderly.

## Supporting Information

S1 Table (SAV)
